# UNRAVELING THE MYSTERY OF VERNIX CASEOSA

**DOI:** 10.4103/0019-5154.41645

**Published:** 2008

**Authors:** Gurcharan Singh, G Archana

**Affiliations:** *From Department of Dermatology and STD, Sri Devaraj Urs Medical College, Tamaka, Kolar - 563 101, India*

**Keywords:** *Emollient*, *postnatal adaptation of skin*, *vernix caseosa*

## Abstract

Vernix caseosa is a white, creamy, naturally occurring biofilm covering the skin of the fetus during the last trimester of pregnancy. Vernix coating on the neonatal skin protects the newborn skin and facilitates extra-uterine adaptation of skin in the first postnatal week if not washed away after birth. It consists of water-containing corneocytes embedded in a lipid matrix. The strategic location of the vernix on the fetal skin surface suggests participation in multiple overlapping functions required at birth, such as barrier to water loss, temperature regulation, and innate immunity. Vernix seems to perform various integral roles during transition of the fetus from intra-uterine to extra-uterine life. It has also found various interesting diagnostic and prognostic implications in this arena. Thus, it continues to be an intriguing topic of interest among the medical fraternity to understand its detailed biology and function in the fetus and also to put its naturally endowed characteristics to use in the adult population.

## Introduction

‘Vernix’ = to varnish. ‘caseous’ = cheesy nature.^1^

Vernix caseosa is a naturally occurring, complex, lipid-rich substance covering the skin surface of the fetus in the last trimester of pregnancy, produced in part by fetal sebaceous glands.^2^,^3^

It provides multiple overlapping maturational functions for the development of skin in utero and adaptation post-birth, reflecting intimate maternal and fetal interactions.

The term ‘Vernix caseosa’ first appeared in 1846 in the Dunglison dictionary of Medical Sciences. Ever since, it has been a fascinating topic of study among the research fraternity and clinicians alike.

This article reviews vernix caseosa, the area of science rich in potential collaborative study, by expert clinicians, as to its fundamental nature, biology, and functions as well as its direct clinical application in skin care.

## Biology of Vernix Caseosa

According to present knowledge, vernix production is unique to humans.

Inherent in the understanding of biology of vernix is a fundamental understanding of the epidermal barrier formation in utero.

As early as 3 weeks of gestation, presumptive epidermis consisting of a single layer of cuboidal cells develops from the embryonic ectoderm^4^ and by the 11^th^ week, the epidermis has three distinctive layers: basal, intermediate, and superficial (periderm).^5^

Beneath the protective cover periderm, the epidermis stratifies and differentiates, forming the four distinctive layers of the epidermis by the end of the 4^th^ month of gestation. Periderm provides a temporary barrier suitable for aqueous environment in utero with active transport mechanism between the amniotic fluid and embryo by virtue of its microvilli at its apical surface. Durable cytoskeletal framework of keratin macro fibrils and cornified envelop is formed to provide mechanical strength analogous to ‘bricks and mortars’.^6^

Periderm cells are replaced continuously until 21 weeks when it is completely shed and replaced by the stratum corneum.^7^ The shed periderm cells are mixed with sebum secretions from the sebaceous glands within the epithelial walls. It is within this combination that vernix caseosa formation occurs.^8^,^9^

An endocrine-based mechanism for vernix production has been proposed.^10^

Hypophyseal- pituitary-adrenal axis regulates sebaceous gland activity of the fetus in utero and subsequently results in production of the superficial lipid film (sebum), first at the vicinity of pilosebaceous unit.^11^–^13^ This changes the transepidermal water gradient and facilitates cornification of the underlying epidermis. Enzymes required in the process, hydroxyl steroid dehydrogenases and 5 alpha-reductase are present after 16 weeks.

The glands reach a peak of activity in the third trimester and their secretion together with desquamated corneocytes into the overlying lipid matrix results in the formation of TRUE VERNIX.

The development of vernix progresses in a cephalocaudal manner and is the result of an orderly progression of epithelial maturation.^14^ It coats the fetus until birth.

Late in the second trimester and particularly in the third trimester, fetal lung maturity parallels with sebaceous gland peak activity and increased physiological concentrations of pulmonary surfactant emulsifies surface vernix.^15^–^17^ There is ‘roll up’ and detachment of vernix and consequent increase in amniotic fluid turbidity.^18^ It is suggested that the coating spreading and detachment of vernix is facilitated by the thermal temperature in utero.^18^

At birth, vernix may cover the entire skin surface or only confined to body folds.^19^ Its color may reflect intra-uterine problems such as hemolytic disease of newborn, post maturity, where it is of golden yellow color. Fetal distress in utero may stain vernix by bile pigments present in meconium.

Very low birth weight infants, i.e., <28 weeks' gestation and < 1000g have very immature and incompetent stratum corneum^20^ and also lack the protective mantle of vernix caseosa. Anecdotal reports indicate that the amount and distribution of vernix on infant at birth are highly variable. Akiba studies^21^ suggest no gender or season effect on vernix coverage and the coverage to be inversely related to birth weight, with maximum for infants fewer than 2000g. Visscher *et al*,^22^ showed that the vernix coverage was higher for lower GA, C-section infants, females, and Caucasian infants and lower following meconium exposure. Coverage was significantly higher on the back than chest, indicating regional differences. Percentage of infants with coverage over entire body surface in these two studies has also been observed to be highly variable.

## Structure and Composition of Vernix Caseosa

Vernix consists of water (81%), lipid (9%), and proteins (10%).^23^

Vernix exhibits a non-lamellar lipid matrix containing hydrated corneocytes with no intercorneal desmosomal connections, in contrast to adult stratum corneum, which contains mature corneocytes and lamellar lipid matrix.^24^

Thus, the vernix structure exhibits “pasta and cheese” morphology with a “mobile” architecture.

Specific material composition reflects cholesterol esters and wax esters, ceramides derived from stratum corneum and sebaceous origin squalene, cholesterol, triglycerides, free fatty acids, phospholipids, and cellular elements.^10^,^25^

Non-polar lipids such as sterol esters and triglycerides predominate among free lipids, having a chain length of up to 32 carbon atoms. The profile of fatty acids [omega]-hydroxyacids and [omega]-hydroxyceramides, representing the bound lipids of vernix shows high similarity to that of stratum corneum; however, vernix lipids show lower degree of ordering as compared to stratum corneum.^26^

Though approximately 80% of vernix is water, it still has high viscosity, suggesting that its water must reside within a highly structured state which hypothetically is conferred by the abundance of water-filled fetal corneocytes in vernix. These fetal corneocytes in utero act as “cellular sponges” to facilitate and maintain cornification by interdicting water moving across the fetal skin, whereas sebaceous lipids in vernix provide a hydrophobic barrier.^27^

Ultra-structural studies show hydrated corneocytes devoid of nuclei and other organelles with sparse network of keratin filaments, about 1-2 micrometers in thickness, lacking desmosomal connections, and surrounded by a thick layer of amorphous lipid without lamellae. Intercellular lipid contains unidentified inclusion bodies, presumably proteinaceous material of keratinocyte origin or sebocyte debris.^27^ Cells in various stages of keratinization may be seen with nuclear remnants.^28^

Histochemistry shows variable acid phosphatase activity intracellularly or in amorphous lipid matrix. Immunofluorescent staining tests of frozen VC smears show that only immunoglobulin G conjugate gives strong positive reaction at antigen sites of VC cells.^28^

Earlier studies had shown that vernix possesses antimicrobial polypeptides. Recently, Maria Tollin *et al*^29^ conducted proteome analysis of vernix caseosa and proved the presence of potent antimicrobial polypeptides. A total of 41 proteins, of which 25 are novel to vernix, have been detected. Effectively, 39% of identified vernix proteins are components of innate immunity and 29% have direct antimicrobial properties.

Origin of vernix proteins seem to be multiple-amniotic fluid, fetal lungs, blood contamination, dermal origins, activated keratinocytes.^29^

## Functions of Vernix Caseosa

### During transition from intra-uterine to extra-uterine life

The vernix within amniotic fluid when swallowed by fetus has potential effects on the developing gut. Glutamine being >20% of amino acid content of vernix is a known trophic factor for the developing gut and is generally required by rapidly proliferating cells such as intestinal epithelium and lymphocytes. Asparginase is also found in abundance, forming >30% of amino acid content.^30^,^31^

Vernix performs an epidermal barrier function in utero to facilitate epidermal growth underneath it and acts as a hydrophobic barrier against amniotic fluid maceration and loss of fluids and electrolytes or TEWL (Trans Epidermal Water Loss).

Vernix also acts as a protective biofilm by minimizing friction of fetal parts during delivery and as an antimicrobial cover against the bacteriologically rich environment of the mother's genital tract along with the insulating effect on the fetus.^32^

### Thermal regulation at birth

Inspite of modern methods of nursing and incubation, the temperature control during the first few hours of life in very low birth weight pre-term infants remains a problematic area in the field of neonatology.

Since these infants have incompetent stratum corneum and high TEWL, it has been suggested by some authors^33^ that the hydrophobic layer of vernix is to be retained after birth and allowed to separate its natural way, which usually occurs by about the 5^th^ day, except in folds of the body where it takes 5 more days to separate. This has shown considerable reduction in the number of cases of subnormal temperature.

However, there is considerable debate on whether vernix has an effect on body temperature regulation and on whether it is to be retained or not. Shulak speculated that vernix could provide thermal stability, but is not a primary factor.^34^ Vernix removal has also been linked to decreased evaporative loss.^35^ The latest study on this issue by Visscher *et al*^22^ has showed no significant effect on thermal regulation by vernix retention. This aspect of function of vernix warrants further studies.

### Skin surface adaptation after birth

Newborn infants undergo a progressive adaptation immediately after birth, including a slow reduction in surface hydration, decrease in skin PH, and stratum corneum dehydration/ desquamation with formation of a dry skin surface. Moreover, there have been regional variations noted in these parameters.^36^,^37^

Vernix may have a role in modulating these processes as evidenced by some studies.

Vernix loses its exogenous water slowly.^27^

Vernix retention after birth results in significantly more hydrated skin surface with higher moisture accumulation rate and higher baseline hydration. This may facilitate postnatal skin hydration.^22^

PH decrease following birth has been attributed to maturation of enzymes responsible for the synthesis of acidic components^37^ and triglycerides in vernix could be a source of acidic fatty acids, provided conditions for hydrolysis are present.^38^

Visccher *et al* suggest that skin surface acidification appears to occur earlier in the presence of vernix retention.^22^

An acidic stratum corneum is believed to inhibit the growth of pathogenic bacteria^39^–^41^ and facilitate colonization with commensal organisms on the skin surface.^42^,^43^

### Antioxidant properties

Vernix is said to have antioxidant properties by virtue of the presence of antioxidants vitamin-E and melanin in it.^44^–^47^

As birth marks a time of high oxidative stress, the antioxidant properties of vernix may help in coping with the pro-oxidant environment as suggested by a decrease in vitamin-E levels in vernix on exposure to ultraviolet light (pro-oxidative stressor).^22^,^48^,^49^

### Anti-infective property

Earlier reports described mechanical barrier properties of vernix with respect to bacterial invasion.^50^ Vernix has also been shown to effectively block penetration of exogenous chymotrypsin present in the amniotic fluid from meconium contamination, while itself being devoid of alpha-chymotryptic activity, while retaining endogenous(epidermal) chymotrypsin.^51^ Recent studies have shown that vernix, like the epidermis, contains antimicrobial peptides and has a direct role in defense against bacteria.^29^,^52^–^54^

Some identified proteins with antimicrobial properties are:^29^

[Alpha]- Defensins [human neutrophil peptide (1-3)]
Cathelicidins (LL-37)
Psoriasin
Ubiquitin
Palate lung nasal epithelial clone (PLUNC)
Neutrophil gelatinase-associated lipocalin (NGAL)
Ribonuclease-7
Annexin 1
Secretory leukocyte protease inhibitors
Calprotectin (calgranulin A, B)

Vernix is also associated with surfactant-associated protein A and surfactant-associated protein D implicated maintenance of airway bacterial homeostasis and also against intra-uterine infection.^55^,^56^

Lysozyme and lactoferrin are the other innate immune proteins present in vernix.^29^

The broad-spectrum action of many of these proteins, in particular the cathelicidins and defensins, may aid in avoiding the development of resistance in bacterial pathogens.^29^

### Moisturizing properties

Because of its high water content, vernix acts as an agent to moisturize the stratum corneum. Comparison with various barrier creams like petrolatum, aquaphor, and eucerin, shows vernix to be having higher water content.^10^

Along with providing “water-proofing” to fetus in utero, it has also been found that application of vernix to adult volar forearm results in an increased capacity to bind exogenous water.^57^

Vernix contains filament aggregating protein, which when broken down forms water-binding molecules referred to as Natural Moisturizing Factor (NMF), which operates to maintain suppleness and plasticity of stratum corneum.^58^

Recent studies have focused on methods to assist with epidermal barrier and evaluating the role of topical emollients in the prevention of infection in pre-term infants.^59^,^60^

However, the emollients lack the active antibacterial properties and the structural barrier-enhancing properties of vernix.

It will not be very long in the future when vernix caseosa may be effectively used as a natural emollient, with all its naturally endowed properties.

### Wound healing properties

Vernix has shown to increase skin metabolism *in vitro* by increasing glucose consumption and lactate production.^61^ The regulation of transepidermal water gradient is known to be important in the epidermal barrier formation and regeneration following wounding;^62^,^63^ and so also the effects of its trophic effects of increased glutamine content. These factors may account for its healing properties in treating adult patients with trophic ulcers of lower extremities^64^ and perineal wounds following delivery.^10^ It may also hence be used in atopic dermatitis against bacterial skin infections.^65^

Given the innate properties of vernix for the neonate-like waterproofing, barrier function, hydration, anti-infective, and antioxidant properties, has benefits for burn patients, who have analogous deficits in skin burn trauma-dehydration and hypovolemia, impaired skin integrity, increased anerobic metabolism, and oxygen-free radicals.^32^

The placement of superficial layer of vernix over laboratory-cultured skin surfaces is currently under investigation, which can then be applied for grafting burn areas.^32^

### Endogenous sking cleansing properties

In experiments performed using human skin soiled with carbon particles, vernix had comparable efficacy to standard commercial skin cleansers.^66^ And unlike commercial soaps, it is capable of providing physiologically relevant lipids to the skin surface with additional moisturization, antioxidation, and infection control, all so important for skin surface integrity.

## Retention of Vernix in Skin Care of Newborn Infant

Traditional practice has directed nurses to wipe vernix caseosa from wet skin of the newborn as part of initial care in the birthing center. As thermoregulation and resuscitation are the priorities of care, wiping of skin was considered the preferred method for accomplishing drying and stimulation of respiratory effort. As the movement toward evidence-based practice has become a major practice effort, the practice and procedure for this nursing activity have fallen under scrutiny.^32^,^67^,^68^ A multiple-site national study was conducted by the National Association of Neonatal Nursing (NANN) and the Association of Women's Health Obstetrical and Neonatal Nursing (AWHONN) in 1998.^69^ A consensus statement based on the results of the study directed “removal of all vernix is not necessary for hygienic reasons” and “vernix may provide antibacterial promotion and wound healing”. Interestingly, the World Health Organization (WHO) also recommends leaving vernix intact on the skin surface after birth.^70^

## Diagnostic and Prognostic Implications of Vernix Caseosa

Identifying elements of vernix caseosa in pulmonary arterial blood using modified version of the procedure originally described by Masson *et al* provides a rapid method of diagnosis of the amniotic fluid embolism following delivery.^71^

A strong association between presence of vernix and mature LSR (Lecithin: Sphingomyelin Ratio) in amniotic fluid (>2) has been seen using an amnioscope. This suggests that amnioscopy may be used to assess fetal maturity before induction of labor, a less invasive procedure than amniocentesis.^72^

Analysis of vernix in the amniotic fluid has been evaluated as a prognostic index of weight of the newborn^73^ and prognostic of maturity status of the fetus.^74^

Vernix caseosa has been used as an alternative to other biological specimens for the determination of fetal cocaine exposure due to its presence in all newborns, ease of collection and storage, and provision of historical record of drug exposure.^75^

Vernix caseosa observed in urine of the parturient, VERNIXURIA, has been reported as an additional sign of uterine rupture.^76^

Vernix caseosa granuloma^77^ and vernix caseosa peritonitis^78^,^79^ have been reported as rare complications of cesarean section, suggesting that in cases in which there is a copious amount of vernix on infants at birth, care should be taken to meticulously irrigate and clear the peritoneal cavity of all debris.

It has been proposed that pregnancy protects against breast cancer, in part, because it results in excretion of lipophilic carcinogens by the mother through fetal fat and vernix caseosa.^80^

The physical properties of vernix hypothetically contribute to the panoply of sensory cues, which attract caregivers to the skin of the newborn; and the possibility of pheromones being a part of vernix is open to investigation.^81^

Vernix caseosa has been implicated in the causation of neonatal aspiration syndrome^82^,^83^ and vernix caseo-granulomatous meningitis.^84^ Hence, pregnant women with a diffuse pattern of high-level echoes in prenatal ultrasonography, suggesting the presence of massive vernix caseosa, should be shifted to a well-equipped institution for delivery.

## References

[1] (1989). The oxford english dictionary.

[2] Hoath SB, Narendran V, Visscher M (2001). Role and biology of vernix. Neonatal Infant Nurs Rev.

[3] Pochi P, Maibach H, Berardesca E (1982). The sebaceous gland. Neonatal skin.

[4] Dietel K, Stave U (1978). Morphological and functional development of the skin. Perinatal physiology.

[5] Moore KL (1988). The developing human: Clinically oriented embryology.

[6] Nemes Z, Steinert PM (1999). Bricks and mortar of the epidermal barrier. Exp Mol Med.

[7] Snell RS (1983). Clinical embryology for medical students.

[8] Pansky B (1982). Medical embryology.

[9] Langman J (1981). Medical embryology.

[10] Narendran V, Hoath SB (2002). The Biology of Vernix Caseosa. J Neonatol.

[11] Holbrook K, Odland G (1980). Regional development of the human epidermis in the first trimester fetus (age related to the timing of amniocentesis and fetal biopsy). J Invest Dermatol.

[12] Holbrook K, Goldsmith L (1991). Structure and function of the developing human skin. Physiology, biochemistry and molecular biology of the skin.

[13] Hardman MJ, Sisi P, Banbury DN, Byrne C (1998). Patterned acquisition of skin barrier function during development. Development.

[14] Elias P (1996). The stratum corneum revisited. J Dermatol.

[15] Pochi PE, Maibach HI, Boisits EK (1982). The sebaceous gland. Neonatal skin.

[16] Holbrook KA, Polin FW, Fox RA (1998). Structural and biochemical organogenesis of skin and cutaneous appendages in the fetus and newborn. Fetal and neonatal physiology.

[17] Froh DK, Ballard PL, Thorburn GD, Harding R (1994). Fetal lung maturation. Textbook of fetal physiology.

[18] Narendran V, Wickett RR, Pickens WL, Hoath SB (2000). Interaction between pulmonary surfactant and vernix: A potential mechanism for induction of amniotic fluid turbidity. Pediatr Res.

[19] Atherton DJ, Gennery AR, Cant AJ, Burns T, Breathnach S, Cox N, Griffiths C The neonate. Rook's Text Book of Dermatology.

[20] Rutter N (1996). The immature skin. Eur J Pediatr.

[21] Akiba T (1955). Studies on biological actions of vernix caseosa. Jpn Obstet Gynecol Soc.

[22] Visscher MO, Narendran V, Pickens WL, LaRuffa AA, Meinzen-Derr J, Allen K (2005). Vernix caseosa in neonatal adaptation. J Perinatol.

[23] Hoeger PH, Schreiner V, Klaassen IA, Enzmann CC, Friedrichs K, Bleck O (2002). Epidermal Barrier lipids in human vernix caseosa: Corresponding ceramide pattern in vernix and fetal skin. Br J Dermatol.

[24] Warner R, Lilly N, Elsner P, Berardesca E, Maibach HI (1994). Correlation of water content with ultrastructure of stratum corneum. Bioengineering of the skin: water and the stratum corneum.

[25] Sumida Y, Yakumaru M, Tokitsu Y (1998). Studies on the function of Vernix caseosa: The secrecy of Baby's skin.

[26] Rissmann R, Groenink HW, Weerheim AM, Hoath SB, Ponec M, Bouwstra JA (2006). New insights into ultrastructure, lipid composition and organization of Vernix Caseosa. J Invest Dermatol.

[27] Pickens W, Warner R, Boissy R, Sb H (2000). Characterization of human vernix: Water content, morphology and elemental analysis. J Invest Dermatol.

[28] Agarastos T, Hollweg G, Grussendorf EI, Papaloucas A (1988). Features of vernix caseosa cells. Am J Perinatol.

[29] Tollin M, Jagerbrink T, Haraldsson A, Agerberth B, Jornvall H (2006). Proteome analysis of vernix caseosa. Pediatr Res.

[30] Baker SM, Balo NN, Abdul Aziz FT (1995). Is Vernix a protective material to the newborn? A biochemical approach. Indian J Pediatr.

[31] Buchman AL (1996). Glutamine: Is it conditionally required nutrient for human gastrointestinal system?. J Am Coll Nutr.

[32] Haubrich RA (2003). Role of vernix caseosa in the neonate. AACN Clin Issues.

[33] Saunders C (1948). The Vernix Caseosa and subnormal temperatures in premature infants. Br J Obstet Gynaecol.

[34] Shaulak B (1963). The antibacterial action of Vernix Caseosa. Harper Hosp Bull.

[35] Riesenfeld B, Strombery B, Sedin G (1984). The influence of Vernix Caseosa on water transport through semi permeable membranes and the skin of full- term infants. Neonatal Physiological Measurements.

[36] Visscher MS, Munson KA, Bare DE, Hoath SB (1999). Early Adaptation of human skin following birth: A biophysical assessment. Skin Res Technol.

[37] Hoeger PH, Enzmann CC (2002). Skin Physiology of the neonate and young infant: A prospective study of functional skin parameters during early infancy. Pediatr Dermatol.

[38] Herrmann F, Behrendt H, Karp F (1946). On the acidity of the surface of the scalp and other areas of the skin in children. J Invest Dermatol.

[39] Puhvel SM, Reisner RM, Amirian DA (1975). Quantification of bacteria in isolated pilosebaceous follicles in normal skin. J Invest Dermatol.

[40] Aly R, Maibach HI, Rahman R, Shinefield HR, Mandel AD (1975). Correlation of human in vivo and in vitro cutaneous antimicrobial factors. J Infect Dis.

[41] Fluhr JW, Kao J, Jain M, Abn SK, Feingold KR, Elias PM (2001). Generation of free fatty acids from phospholipids regulates stratum corneum acidification and integrity. J Invest Dermatol.

[42] Parra JL, Paye M (2003). EEMCO guidance for the in vivo assessment of skin surface PH. Skin Pharmacol Appl Skin Physiol.

[43] Lukacs A, Brain-Falco O, Korting H (1992). Invitro control of the growth of important bacteria of the resident flora by changes in PH. Skin cleansing with synthetic detergents.

[44] Thiele J, Weber S, Packer L (1999). Sebaceous gland secretion is a major physiological route of Vitamin E delivery to skin. J Invest Dermatol.

[45] Thiele J, Packer L (1999). Non-invasive measurement of alpha-tocopherol gradients in human stratum corneum by high performance liquid chromatography analysis of sequential tape strippings. Met Enzymol.

[46] Pickens W, Zhou Y, Wickett R, Vischer M, Hoath S (2000). Antioxidant defense mechanisms in Vernix Caseosa: Potential role of endogenous Vitamin E. Pediatr Res.

[47] Youssef W, Hoath S (2001). Surface free energy characterization of Vernix caseosa: Role in waterproofing the newborn infant. Skin Res Technol.

[48] Thiele JJ, Traber MG, Polefka TG, Cross CE, Packer L (1997). Ozone-exposure depletes Vitamin E and induces lipid perioxidation in murine stratum corneum. J Invest Dermatol.

[49] Thiele JJ, Traber MG, Packer L (1998). Depletion of human stratum corneum Vitamin E: An early and sensitive in vivo marker of UV induced photooxidation. J Invest Dermatol.

[50] Joglekar VM (1980). Barrier properties of Vernix Caseosa. Arch Dis Child.

[51] Tansirikongkol A, Wickett RR, Visscher MO, Hoath SB (2007). Effect of Vernix Caseosa on the penetration of chymotryptic enzyme: Potential role in epidermal Barrier development. Pediatr Res.

[52] Yoshio H, Tollin M, Gudmundsson GH, Lagercrantz H, Jornvall H, Marchini G (2003). Antimicrobial polypeptides of human Vernix Caseosa and amniotic fluid: Implications for newborn innate defense. Pediatr Res.

[53] Tollin M, Bergsson G, Kai-Larsen Y, Lengqvist J, Sjövall J, Griffiths W (2005). Vernix Caseosa as a multi component defense system based on polypeptides, lipids and their interactions. Cell Mol Life Sci.

[54] Akinbi HT, Narendran V, Pass AK, Markart P, Hoath SB (2004). Host defense proteins in Vernix Caseosa and amniotic fluid. Am J Obstet Gynecol.

[55] Narendran V, Hull W, Akinbi H (2000). Vernix Caseosa contains surfactant proteins: Potential Role in innate immune function in the fetus. Pediatr Res.

[56] Kuroki Y, Sano H (1999). Functional roles and structural analysis of lung collectins SP-A and SP-D. Biology Neonate.

[57] Bautista MI, Wickett RR, Vischer MO, Pickens WL, Hoath SB (2000). Characterization of Vernix Caseosa as a natural biofilm: Comparison to standard oil based ointments. Pediatr Dermatol.

[58] Rawlings AV, Scott IR, Harding CR, Bowser PA (1994). Stratum corneum moisturization at the molecular level. J Invest Dermatol.

[59] Nopper AJ, Horii KA, Sookdeo-Drost S, Wang TH, Mancini AJ, Lane AT (1996). Topical ointment therapy benefits premature infants. J Pediatr.

[60] Conner JM, Soll RF, Edward WH (2004). Topical ointment for preventing infection in preterm infants. Cochrane Database Syst Rev.

[61] Barai N (2005). Effect of Vernix Caseosa on epidermal barrier development-repair: Implications in wound healing. College of Pharmacy.

[62] Grubauer G, Elias P, Feingold K (1989). Transepidermal water loss: The signal for recovery of barrier structure and function. J Lipid Res.

[63] Proksch E, Holleran WM, Menon GK, Elias PM, Feingold KR (1993). Barrier function regulates epidermal lipid and DNA synthesis. Br J Dermatol.

[64] Zhukov B, Neverova E, Nikitin K (1992). A comparative evaluation of the use of Vernix Caseosa and Solcoseryl in treating patients with trophic ulcers of lower extremities. Vestnik Khirurgii Imeni I I Grekova.

[65] Roos TC, Geuer S, Roos S, Brost H (2004). Recent advances in treatment strategies for Atopic Dermatitis. Drugs.

[66] Morailli R, Pickens WL, Vischer MO, Hoath SB (2004). A novel role for Vernix Caseosa as a skin cleanser. Biol Neonate.

[67] Lund C, Kuller J, Lane A, Lott JW, Raines DA (1999). Neonatal skin care: The scientific basis for practice. Neonatal Netw.

[68] Lott J, Hoath S (1998). Neonatal skin: The ideal nursing interface. J Pediatr Nurs.

[69] Association of Women's Health Obstetrical and Neonatal Nurses (AWHONN) and National Association of Neonatal Nursing (NANN) (2001). Evidence-based nursing practice-skin care.

[70] Hoath SB, Narendran V (2004). 50 years ago in The Journal of Pediatrics. J Pediatr.

[71] Dolyniuk M, Orfei E, Vania H, Karlman R, Tomich P (1983). Rapid diagnosis of amniotic fluid embolism. Obstet Gynecol.

[72] McLaughlan JM, Chang AM (1977). Lecithin: Sphingomyelin ratio and presence of vernix in amniotic fluid.

[73] Varela J, Pettinelli E, Molina R, Naverrete J, Zambrano N, Vera R (1974). Analysis of the vernix in the amniotic fluid as a prognostic index of the weight of the newborn. Rev Chil Obstet Ginecol.

[74] Sepulveda WH, Araneda H, Avendano A (1991). Ultrasonic detection of fetal vernix in amniotic fluid as an index to fetal maturity. Rev Chil Obstet Ginecol.

[75] Moore C, Dempsey D, Deitermann D, Lewis D, Leikin J (1996). Fetal cocaine exposure: Analysis of vernix caseosa. J Anal Toxicol.

[76] O'Grady JP, Prefontaine M, Hoffman DE (2003). Vernixuria: Another sign of uterine rupture. J Perinatol.

[77] Boothby R, Lammert N, Benrubi GI, Weiss B (1985). Vernix caseosa granuloma: A rare complication of cesarean section. Southern Med J.

[78] Krunerman MS, Pouliot GJ (1976). Maternal vernix caseosa peritonitis: Rare complications of cesarean sections. N Y State J Med.

[79] Mahmoud A, Silapaswas S, Lin K, Penney D (1997). Vernix caseosa: An unusual cause of post-cesarean section peritonitis. Am Surg.

[80] Levine RS, Dolin P (1992). Pregnancy and breast cancer: A possible explanation for the negative association. Med Hypotheses.

[81] Hoath SB, Pickens WL, Visscher MO (2006). The biology of vernix caseosa. Int J Cosmet Sci.

[82] Nishijima K, Shukunami K, Inoue S, Kotsuji F (2005). Management for neonatal aspiration syndrome caused by vernix caseosa. Fetal Diagn Ther.

[83] Ohlsson A, Cumming WA, Najjar H (1985). Neonatal aspiration syndrome due to vernix caseosa. Pediatr Radiol.

[84] Midha R, Becker LE (1991). Vernix caseo granulomatous meningitis (vernicomyelia). Can J Neurol Sci.

